# Agreement of consumer wearable pulse oximeters across activity conditions and skin pigmentation

**DOI:** 10.3389/fdgth.2026.1909802

**Published:** 2026-10-30

**Authors:** Kailey Miller, Hayley Whitney, Natalia Chavez, Dhruv R. Seshadri

**Affiliations:** 1 Department of Mechanical Engineering, Lehigh University, Bethlehem, PA, United States; 2 Department of Bioengineering, Lehigh University, Bethlehem, PA, United States; 3 College of Health, Lehigh University, Bethlehem, PA, United States

**Keywords:** pulse oximetry, oxygen saturation, skin pigmentation, wearable sensors, photoplethysmography, measurement agreement, physical activity

## Abstract

**Introduction:**

Wearable pulse oximetry devices are increasingly used in consumer-grade health products and patient monitoring; however, concerns remain regarding their accuracy across diverse populations and non-ideal conditions.

**Methods:**

This study evaluated the agreement of four consumer wearable pulse oximeters with two clinical comparator monitors in a cohort of 49 adults during seated rest, treadmill walking, and seated recovery conditions. Skin tone was assessed using both the visual Fitzpatrick scale and the Individual Typology Angle (ITA), derived from objective skin color measurements. Device performance was evaluated using signed and absolute measurement error, generalized estimating equations accounting for repeated measurements, and repeated-measures Bland–Altman analysis.

**Results:**

Agreement varied by device and activity condition. Garmin showed a significant negative signed bias relative to the GE Dash during cooldown (−0.72 percentage points, 95% CI −1.19 to −0.25; Holm-adjusted *p* = 0.028), while Wellue showed a significant negative signed bias during treadmill activity (−1.03 percentage points, 95% CI −1.52 to −0.53; Holm-adjusted *p* < 0.001). Wellue also demonstrated significant condition-dependent changes between treadmill and cooldown for both signed and absolute error. No device-specific association between skin pigmentation and signed or absolute error remained significant after correction for multiple comparisons using either continuous ITA or Fitzpatrick grouping. Measurement availability was substantially reduced for Garmin during treadmill activity (82.4%) and was nearly absent for the Apple Watch during treadmill activity (0.4%).

**Discussion:**

These findings demonstrate that agreement between wearable and clinical pulse oximeters varies by device and activity state, while no statistically significant pigmentation-associated effects were identified after correction for multiple comparisons. Incorporating objective skin tone metrics, diverse participant populations, and activity-aware evaluation protocols will be essential for improving device reliability, supporting equitable digital health technologies, and reducing disparities in wearable-based physiological monitoring.

## Introduction

Blood oxygen saturation (SpO₂) is a key physiological metric used to assess respiratory and cardiovascular status and to guide clinical decision-making across a wide range of conditions, including chronic obstructive pulmonary disease (COPD), obstructive sleep apnea, acute respiratory infections, and COVID−19 (1–3). Accurate detection of hypoxemia is essential for timely intervention and escalation of care, particularly in both acute and chronic disease management. Advances in optical sensing and device miniaturization have enabled SpO₂ monitoring to extend beyond clinical environments into consumer wearable devices, allowing continuous, real-world assessment of oxygenation outside of supervised care settings (4–6). As these devices are increasingly incorporated into health tracking, telemedicine, and remote patient monitoring, their measurement performance and reliability have become matters of growing clinical and public health importance (7, 8).

Despite widespread adoption, the performance of wearable SpO₂ devices has not been comprehensively evaluated across diverse user populations or real-world usage conditions. Wearable oximetry relies on photoplethysmography (PPG), which estimates arterial oxygen saturation using wavelength-dependent light absorption in biological tissue (9). Although PPG-based sensing is widely used due to its low power consumption and suitability for compact devices, its measurement performance is sensitive to multiple factors, including sensor design, anatomical placement, motion artifacts, peripheral perfusion, and tissue optical properties (7, 9, 11, 12). Differences in device architecture, such as reflective sensors used in wrist-worn wearables versus transmissive sensors used in finger-based oximeters, may further contribute to variability in signal quality, particularly during physical activity (9, 13). In addition, individual physiological characteristics, including age and body mass index (BMI), may influence vascular dynamics and tissue composition, yet these factors are rarely examined together within a unified evaluation framework for consumer SpO₂ devices (11).

In parallel with experimental evaluation studies, computational modeling has become an increasingly important tool for understanding the optical mechanisms underlying pulse oximetry performance. Among these approaches, Monte Carlo light-transport simulations are commonly used to model photon propagation through biological tissues because they enable controlled evaluation of tissue optical properties that are difficult or impossible to isolate experimentally. By simulating interactions among epidermal melanin, dermal blood volume, tissue scattering, sensor geometry, and illumination wavelengths, these models provide insight into how device design influences measurement performance before clinical testing. Increasing epidermal melanin can reduce detected photon intensity and alter pulse modulation at wavelengths commonly used in pulse oximetry, including approximately 660 nm (red) and 940 nm (infrared). These pigmentation-dependent optical changes may alter the ratio-of-ratios used to estimate arterial oxygen saturation, shift device calibration curves, and contribute to systematic measurement bias, particularly for reflective photoplethysmography systems used in consumer wearable devices. For example, Moradi et al. demonstrated that variations in skin pigmentation and device geometry substantially influence light transport and pulse-oximetry signal formation, highlighting the importance of considering tissue optics alongside experimental device evaluation when assessing wearable SpO₂ performance (21, 22).

Although computational modeling predicts pigmentation-dependent changes in optical signal propagation, relatively few experimental studies have systematically evaluated whether these effects translate into differences in wearable pulse oximeter performance under real-world conditions. Consequently, skin pigmentation remains an underexplored source of variability in wearable SpO₂ measurement. Melanin absorbs light across both the visible and near-infrared spectrum, overlapping the wavelengths used in pulse oximetry and potentially altering optical signal propagation through tissue (14, 15). Consistent with predictions from Monte Carlo light-transport models, increased epidermal melanin may reduce detected photon intensity and pulse amplitude, potentially affecting signal-to-noise characteristics and SpO₂ estimation, particularly under conditions of motion or reduced peripheral perfusion (10, 15). While recent clinical studies have raised concerns about pigmentation-related bias in pulse oximetry, particularly in hospital settings, there remains a lack of systematic, device-level evaluation studies examining whether wearable SpO₂ agreement differs across skin tones, especially in consumer devices and during physical activity (1, 16, 17). Many existing wearable evaluations either do not report skin tone or rely on limited and demographically homogeneous cohorts, restricting generalizability and obscuring potential sources of bias. When skin tone is considered in biomedical research, it is often characterized subjectively, such as self-identified race or categorical dermatological scales. The Fitzpatrick skin type scale remains widely used in clinical and device studies but was not designed to capture optical properties relevant to light-based biosensing (18). Objective, quantitative approaches, such as the Individual Typology Angle (ITA), derived from L*b* color space measurements, provide a continuous and physiologically meaningful representation of skin pigmentation and have been validated in recent dermatological and optical research (14, 19). However, objective skin tone metrics remain rarely incorporated into wearable SpO₂ evaluation studies, representing an important area for further investigation.

Inaccurate SpO₂ measurements may reduce confidence in wearable-derived oxygen saturation estimates and contribute to measurement variability across different patient populations. Because wearable pulse oximeters are increasingly incorporated into remote monitoring and digital health applications, understanding the factors that influence measurement agreement is important for improving device performance and promoting equitable health technology (2, 3, 16). As wearable sensor data increasingly influence clinical workflows, population-level health analytics, and algorithm-driven decision systems, unrecognized performance variability across skin tones raises concerns regarding the reliability and equitable use of wearable-derived physiological data (17, 20). Without rigorous agreement testing across diverse populations, technologies intended to democratize health monitoring risk perpetuating or exacerbating existing disparities in care.

Although computational modeling provides important mechanistic insight into light-tissue interactions, experimental studies are needed to determine whether these predicted effects are associated with differences in the performance of commercially available wearable pulse oximeters during real-world use. Therefore, the primary objective of this study was to evaluate the agreement between consumer wearable pulse oximeters and clinical pulse oximeters across varying activity conditions and skin pigmentation. By examining device performance in a cohort spanning a broad range of objectively measured skin pigmentation, this study aims to contribute evidence supporting the development of more equitable and reliable wearable health technologies.

## Materials and methods

A total of 49 adult participants were enrolled and included in the final analysis. The cohort consisted of 23 males and 26 females. All participants were 18 years of age or older and capable of completing light treadmill exercise. Exclusion criteria included age under 18 years, presence of a pacemaker, known cardiac rhythm disorders, diagnosed respiratory disorders, or tattoos at device placement sites. Participants with diagnosed respiratory disorders were excluded to minimize confounding from underlying impairments in oxygenation and to ensure participant safety during treadmill exercise. Restricting the study to healthy participants allowed device performance to be evaluated under standardized physiological conditions. Participant characteristics, including age, biological sex, body mass index (BMI), and skin tone classification, were collected prior to data acquisition. Biological sex (male or female) was self-reported by each participant during study enrollment. Summary statistics for age, BMI, ITA, and Fitzpatrick skin type are provided in Table 1, with Fitzpatrick and descriptive ITA category distributions presented in Table 2. The sample size was determined based on feasibility and the goal of obtaining representation across a broad range of skin pigmentation levels while collecting repeated measurements across multiple consumer and clinical pulse oximeters under standardized testing conditions. The study protocol was approved by the Lehigh University Institutional Review Board (STUDY #2237852−5), and all participants provided written informed consent prior to participation.

**Table 1:** Summary statistics for age, body mass index (BMI), Individual Typology Angle (ITA), and Fitzpatrick scale among participants included in the final analysis. Values are reported as minimum, maximum, mean ± standard deviation (SD), and interquartile ranges expressed as the first and third quartiles (Q1-Q3), representing the 25th and 75th percentiles, respectively.

Variable	Minimum	Maximum	Mean ± SD	IQR (Q1–Q3)
Age (years)	18.0	72.0	25.5 ± 10.7	[19.0–27.0]
BMI (kg/m^2^)	16.4	39.5	24.5 ± 4.2	[21.4–26.4]
ITA	−90.0	59.0	−8.9 ± 42.7	[−47.0–29.0]
Fitzpatrick scale	1.0	6.0	3.4 ± 1.3	[3.0–4.0]

**Table 2:** Fitzpatrick skin type and descriptive ITA category distribution among participants included in the final analysis (*N* = 49).

Fitzpatrick Skin Type		ITA Category	
Type	n (%)	Category	n (%)
I	1 (2.0)	Dark	17 (34.7)
II	11 (22.4)	Tan	13 (26.5)
III	18 (36.7)	Intermediate	5 (10.2)
IV	8 (16.3)	Light	9 (18.4)
V	7 (14.3)	Very Light	3 (6.1)
VI	4 (8.2)	Extremely Light	2 (4.1)

SpO₂ measurements were collected using six pulse oximetry devices, including four consumer devices, the Garmin Fenix 7 smartwatch (Garmin), Apple Watch Series 6 (Apple Watch), Wellue O2Ring Wearable Oxygen Monitor (Wellue), and Zacurate 500BL Finger Pulse Oximeter (Zacurate), and two clinical comparator devices, the Welch Allyn Vital Signs Monitor 6000 Series (Welch Allyn) and GE Dash 3000 Monitor (GE Dash 3000). Both clinical comparator monitors were operated using their manufacturer-default SpO₂ settings, with no device settings modified for the study. Device-specific sensor model, firmware version, and installed SpO₂ module configuration were not recorded prospectively. Wrist-worn devices used reflective photoplethysmography sensors, while finger-based devices used transmissive sensor configurations (Figure 1).

**Figure 1:**
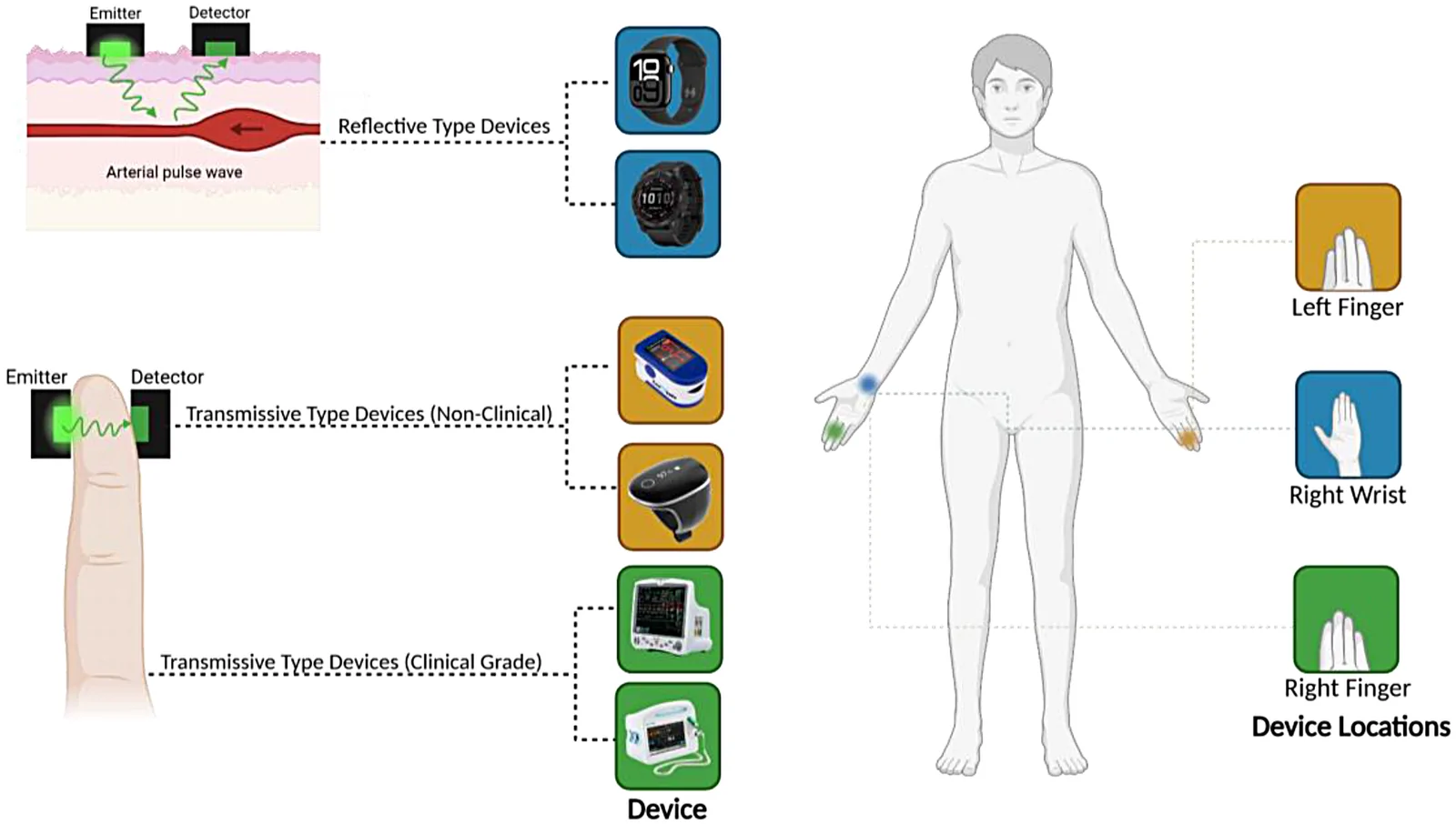
Overview of device types and placement used during SpO₂ testing. Reflective-type devices were worn on the wrist, with the emitter and detector on the same side of the skin, while transmissive-type devices were placed on the finger, with the emitter and detector on opposite sides. The figure includes both consumer devices and clinical comparator devices and shows device placement locations for each round of testing, color-coded by anatomical site: wrist (blue), left finger (green), and right finger (orange). Created in BioRender. Lab, S. (2026) https://BioRender.com/y67o0fj.

The Welch Allyn and GE Dash 3000 systems were selected as clinically comparator devices because they are routinely used in hospital settings for continuous pulse oximetry monitoring. Although arterial blood gas co-oximetry remains the reference standard for measuring arterial oxygen saturation (SaO₂), its invasive nature makes it impractical for repeated measurements during treadmill exercise in healthy participants. Accordingly, this study evaluated agreement between consumer wearable devices and clinical pulse oximeters, rather than validation of absolute SpO₂ accuracy against arterial co-oximetry.

The Apple Watch Series 6 provided on-demand SpO₂ measurements through the Blood Oxygen application and operated on watchOS 11.5 during data collection. During each scheduled measurement, participants initiated an SpO₂ reading using the Blood Oxygen application while wearing the device on the wrist. The application requires the wearer to remain stationary during measurement. Because the study protocol required participants to continue walking during treadmill data collection, successful Apple Watch SpO₂ acquisition was limited during this condition. Consequently, Apple Watch treadmill measurements were treated as unavailable when no valid SpO₂ value was produced rather than as physiologic measurements.

Participants completed a standardized protocol consisting of three sequential activity conditions: seated rest, treadmill walking, and seated recovery. During each condition, SpO₂ and heart rate (HR) values were recorded once per minute for five consecutive minutes. Participants were first instrumented with the Garmin Fenix 7, Wellue finger pulse oximeter, and GE Dash 3000 clinical comparator. While seated, measurements were collected at one-minute intervals for five minutes. Participants then walked on a treadmill at a fixed speed of 2.5 miles per hour for five minutes, with measurements recorded at one-minute intervals. Following treadmill activity, participants returned to a seated position for a five-minute cooldown period, during which measurements continued at one-minute intervals. After completion of the first device set, participants were given a one-minute rest period to allow for device removal and placement of the second device set. The protocol was then repeated using the Apple Watch Series 6, Zacurate finger pulse oximeter, and Welch Allyn clinical comparator. Device order was fixed across participants. At each one-minute time point, the researcher verbally indicated the measurement interval, and participants read their displayed SpO₂ and HR values aloud from each device.

Participants completed the protocol in two sequential testing rounds because all six devices could not be worn simultaneously without interfering with sensor placement. During the first testing round, participants wore the Garmin Fenix 7, GE Dash 3000, and Wellue pulse oximeter while completing five minutes of seated rest, five minutes of treadmill walking (2.5 mph), and five minutes of seated recovery. Following completion of the first round, the devices were removed, participants rested for approximately one minute to allow for device exchange, and the protocol was repeated using the Apple Watch Series 6, Welch Allyn monitor, and Zacurate pulse oximeter under identical testing conditions. Device order was fixed for all participants to maintain consistent sensor placement, minimize variability associated with repeated device application, and standardize the testing procedure across participants. Because testing order was not randomized, potential order-related effects could not be separated from differences between the two testing rounds and were therefore considered a study limitation. Direct comparisons were performed only between consumer devices and the clinical comparator monitor measured during the same testing round (Garmin and Wellue vs. GE Dash during Round 1; Apple Watch and Zacurate vs. Welch Allyn during Round 2). Devices measured in different testing rounds were not directly compared because measurements were not acquired simultaneously.

Values were documented manually in a structured Excel spreadsheet in real time. Data collection was conducted in an indoor laboratory environment under consistent ambient lighting conditions. Skin tone was assessed using both subjective and objective approaches. Subjective classification was performed using the Fitzpatrick skin type scale, while objective skin pigmentation was quantified using CIE L* and b* color-space measurements to calculate the Individual Typology Angle (ITA°). ITA values were used to stratify participants into standardized skin tone categories ranging from very light to dark (Figure 2).

**Figure 2:**
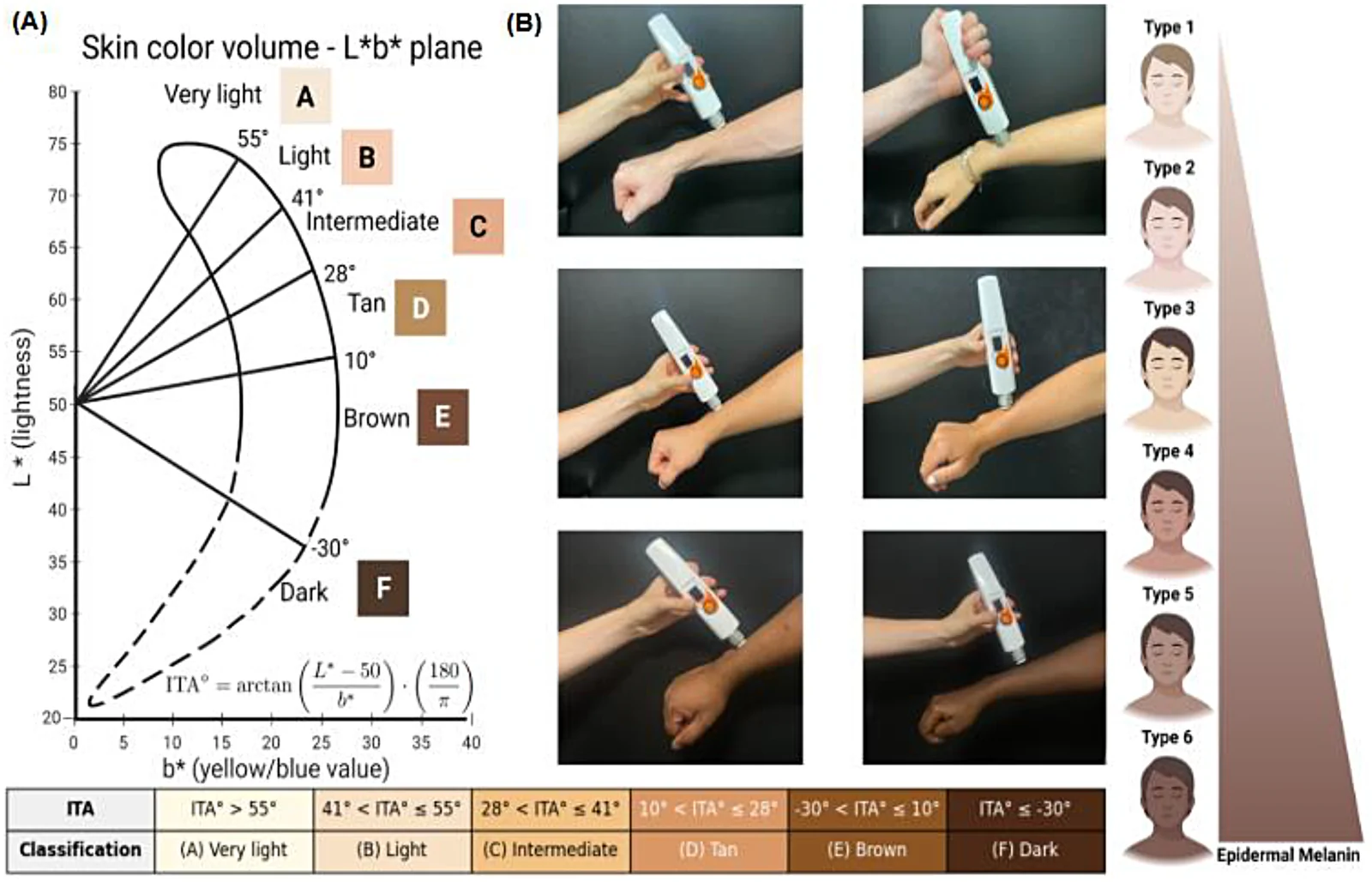
Skin tone classification using the L*b* color space and individual typology angle (ITA°). **(A)** shows a skin color volume chart with ITA-based boundaries dividing six tone categories **(A–F)** on the L* (lightness) and b* (yellow-blue) axes. **(B)** displays representative forearms illustrating six skin-tone classifications, each measured with a handheld device. Created in BioRender. Lab, S. (2026) https://BioRender.com/gof55i3.

**Table 3:** Measurement availability, missingness, and exclusions by device and activity condition. Excluded measurements represent scheduled consumer-device observations that could not be included in synchronized consumer–comparator analyses because the consumer-device SpO₂ measurement was missing. No clinical-comparator measurements were missing. Apple Watch treadmill missingness primarily reflected the device's requirement for the wearer to remain stationary during on-demand SpO₂ measurement.

Device	Condition	Expected	Recorded	Missing, *n* (%)	Excluded	Availability (%)
Garmin	Sitting	245	245	0 (0.0)	0	100.0
Garmin	Treadmill	245	202	43 (17.6)	43	82.4
Garmin	Cooldown	245	241	4 (1.6)	4	98.4
Wellue	Sitting	245	245	0 (0.0)	0	100.0
Wellue	Treadmill	245	245	0 (0.0)	0	100.0
Wellue	Cooldown	245	245	0 (0.0)	0	100.0
GE Dash 3000	Sitting	245	245	0 (0.0)	0	100.0
GE Dash 3000	Treadmill	245	245	0 (0.0)	0	100.0
GE Dash 3000	Cooldown	245	245	0 (0.0)	0	100.0
Apple Watch	Sitting	245	244	1 (0.4)	1	99.6
Apple Watch	Treadmill	245	1	244 (99.6)	244	0.4
Apple Watch	Cooldown	245	245	0 (0.0)	0	100.0
Zacurate	Sitting	245	245	0 (0.0)	0	100.0
Zacurate	Treadmill	245	245	0 (0.0)	0	100.0
Zacurate	Cooldown	245	245	0 (0.0)	0	100.0
Welch Allyn	Sitting	245	245	0 (0.0)	0	100.0
Welch Allyn	Treadmill	245	245	0 (0.0)	0	100.0
Welch Allyn	Cooldown	245	245	0 (0.0)	0	100.0

Skin tone was assessed using both subjective and objective methods. Subjective skin tone classification was assigned by a trained researcher using the Fitzpatrick Skin Type scale prior to the commencement of SpO₂ data collection. Objective skin pigmentation was measured using the SkinColorCatch colorimeter (Delfin Technologies Ltd., Kuopio, Finland). The instrument was calibrated according to the manufacturer's instructions prior to data collection. A single measurement was obtained from the participant's right wrist under consistent indoor laboratory fluorescent lighting conditions. The instrument recorded CIE L* and b* color-space values, which were subsequently used to calculate the Individual Typology Angle (ITA°). ITA was calculated as $ITA^{\circ }=\frac{180}{\pi }\tan^{-1}\;\left(\frac{L^{*}-50}{b^{*}}\right)$, where L* represents lightness and b* represents the yellow–blue color coordinate.

Participants were then classified into standardized ITA skin-tone categories ranging from very light to dark. Skin pigmentation measurements were collected before physiological testing and before review of device-performance results to minimize the potential for measurement-related bias.

For each device and activity condition, the number of expected, successful, missing, and excluded SpO₂ measurements was recorded. Missing measurements were defined as scheduled observations for which no valid SpO₂ value was available at the designated time point. For the consumer–comparator agreement analyses, only synchronized measurement pairs in which both the consumer device and corresponding clinical comparator produced a valid SpO₂ value were included. Pairwise observations were excluded when the consumer-device measurement was missing; no clinical-comparator measurements were missing. No imputation was performed for missing SpO₂ values.

### Statistical analysis

Continuous variables were summarized as mean ± standard deviation, and categorical variables as counts and percentages. For each synchronized consumer–comparator measurement pair, signed error was calculated as consumer-device SpO₂ minus comparator SpO₂, and absolute error as the absolute value of this difference.

Repeated measurements were analyzed using generalized estimating equations (GEE) with a Gaussian distribution, exchangeable correlation structure, and participant-level clustering. Separate models were fit for each testing round and included device, activity condition, continuous ITA, and their interaction terms. Device- and condition-specific contrasts were used to evaluate differences in signed and absolute error. Fitzpatrick skin type, grouped as I–II, III–IV, and V–VI, was evaluated as a secondary measure of skin pigmentation. Holm correction was applied to multiple comparisons. Sensitivity analyses additionally adjusted for age, BMI, and sex.

Agreement between each consumer device and its corresponding clinical comparator was further evaluated using repeated-measures Bland–Altman analysis and intraclass correlation coefficients (ICC) (23, 24). Bland–Altman analyses estimated mean bias and 95% limits of agreement while accounting for repeated observations within participants. ICCs were calculated separately for each device and activity condition using participant-level mean SpO₂ values across the five measurement time points. A two-way mixed-effects, absolute-agreement, single-measure ICC was used, with 95% confidence intervals reported. Apple Watch treadmill agreement was not evaluated because only one valid synchronized measurement was available.

Statistical significance was defined as two-sided *p* < 0.05. Analyses were performed in Python 3.12.13.

## Results

### Participant characteristics

A total of 49 participants were included in the final analysis, including 26 females (53.1%) and 23 males (46.9%). Mean age was 25.5 ± 10.7 years, mean BMI was 24.5 ± 4.2 kg/m^2^, and mean Individual Typology Angle (ITA) was −8.9 ± 42.7°, with values ranging from −90° to 59° (Table 1). Fitzpatrick skin types were distributed as follows: type I, 1 (2.0%); type II, 11 (22.4%); type III, 18 (36.7%); type IV, 8 (16.3%); type V, 7 (14.3%); and type VI, 4 (8.2%) (Table 2). For secondary grouped analyses, 12 participants (24.5%) were classified as Fitzpatrick I–II, 26 (53.1%) as III–IV, and 11 (22.4%) as V–VI (Table 1).

### Measurement availability and missingness

Across the six devices, three activity conditions, and five scheduled measurements per condition, 4,410 device-level SpO₂ readings were expected and 4,118 were successfully recorded, resulting in 292 missing readings (6.6%). For consumer–clinical comparator analyses, 2,940 synchronized measurement pairs were expected and 2,648 valid pairs were available for analysis. All 292 excluded pairwise observations resulted from missing consumer-device measurements; no clinical-comparator measurements were missing (Table 3).

Measurement availability varied by device and activity condition. The GE Dash 3000, Welch Allyn, Wellue, and Zacurate produced 100% of expected measurements across all three activity conditions. Garmin produced all expected measurements during sitting, 202 of 245 treadmill measurements (82.4%), and 241 of 245 cooldown measurements (98.4%). Apple Watch availability was 99.6% during sitting (244/245) and 100% during cooldown (245/245) but was nearly absent during treadmill activity, with only 1 of 245 expected readings recorded (0.4%). During treadmill activity, 48 of 49 participants had no Apple Watch SpO₂ readings (Table 3).

### Agreement with clinical comparators

Adjusted agreement estimates differed by device and condition (Table 4). Mean SpO₂ measurements over time across activity conditions are presented in Figure 3. Holm-adjusted tests of signed bias are summarized separately in Table 5. Garmin showed a significant negative signed bias relative to the GE Dash 3000 during cooldown of −0.72 percentage points (95% CI −1.19 to −0.25; Holm-adjusted *p* = 0.028). Garmin treadmill bias was −0.47 percentage points (95% CI −0.93 to −0.02) but was not significant after correction for multiple comparisons (Holm-adjusted *p* = 0.324). Wellue showed a significant negative signed bias relative to the GE Dash 3000 during treadmill activity of −1.03 percentage points (95% CI −1.52 to −0.53; Holm-adjusted *p* < 0.001). Signed bias during sitting and cooldown were not statistically significant after correction. Apple Watch signed bias relative to Welch Allyn was not statistically significant during sitting or cooldown. Apple Watch treadmill agreement was not estimated because only one valid synchronized treadmill measurement was available. Zacurate showed no statistically significant signed bias relative to Welch Allyn after correction for multiple comparisons across the three activity conditions (Table 4).

**Table 4:** Agreement between consumer pulse oximeters and their corresponding clinical comparators across activity conditions. Garmin and Wellue were compared with the GE Dash 3000, while Apple Watch and Zacurate were compared with the Welch Allyn. Signed bias represents consumer-device SpO₂ minus comparator SpO₂; absolute error represents the absolute value of this difference.

Device	Condition	N	Signed bias (95% CI)	Absolute error (95% CI)
*GE Dash 3000 comparator*				
Garmin	Sitting	245	−0.17 (−1.44, 1.09)	2.59 (1.47, 3.71)
Garmin	Treadmill	202	−0.47 (−0.93, −0.02)	1.64 (1.38, 1.90)
Garmin	Cooldown	241	−0.72 (−1.19, −0.25)	1.79 (1.50, 2.09)
Wellue	Sitting	245	0.77 (−0.42, 1.97)	2.31 (1.18, 3.43)
Wellue	Treadmill	245	−1.03 (−1.52, −0.53)	2.04 (1.67, 2.41)
Wellue	Cooldown	245	0.34 (−0.05, 0.74)	1.42 (1.19, 1.65)
*Welch Allyn comparator*				
Apple Watch	Sitting	244	0.19 (−0.22, 0.60)	1.64 (1.45, 1.84)
Apple Watch	Treadmill	1	Not estimable	Not estimable
Apple Watch	Cooldown	245	−0.27 (−0.68, 0.14)	1.62 (1.39, 1.84)
Zacurate	Sitting	245	0.38 (0.04, 0.72)	1.21 (1.02, 1.40)
Zacurate	Treadmill	245	−0.35 (−0.81, 0.12)	1.82 (1.40, 2.23)
Zacurate	Cooldown	245	0.09 (−0.29, 0.46)	1.35 (1.13, 1.57)

**Table 5:** Holm-adjusted tests of signed bias between consumer devices and their clinical comparators.

Device	Condition	Holm-adjusted p
Garmin	Sitting	1.000
Garmin	Treadmill	0.324
Garmin	Cooldown	0.028
Wellue	Sitting	1.000
Wellue	Treadmill	<0.001
Wellue	Cooldown	0.612
Apple Watch	Sitting	1.000
Apple Watch	Treadmill	Not estimable
Apple Watch	Cooldown	1.000
Zacurate	Sitting	0.249
Zacurate	Treadmill	0.847
Zacurate	Cooldown	1.000

**Figure 3:**
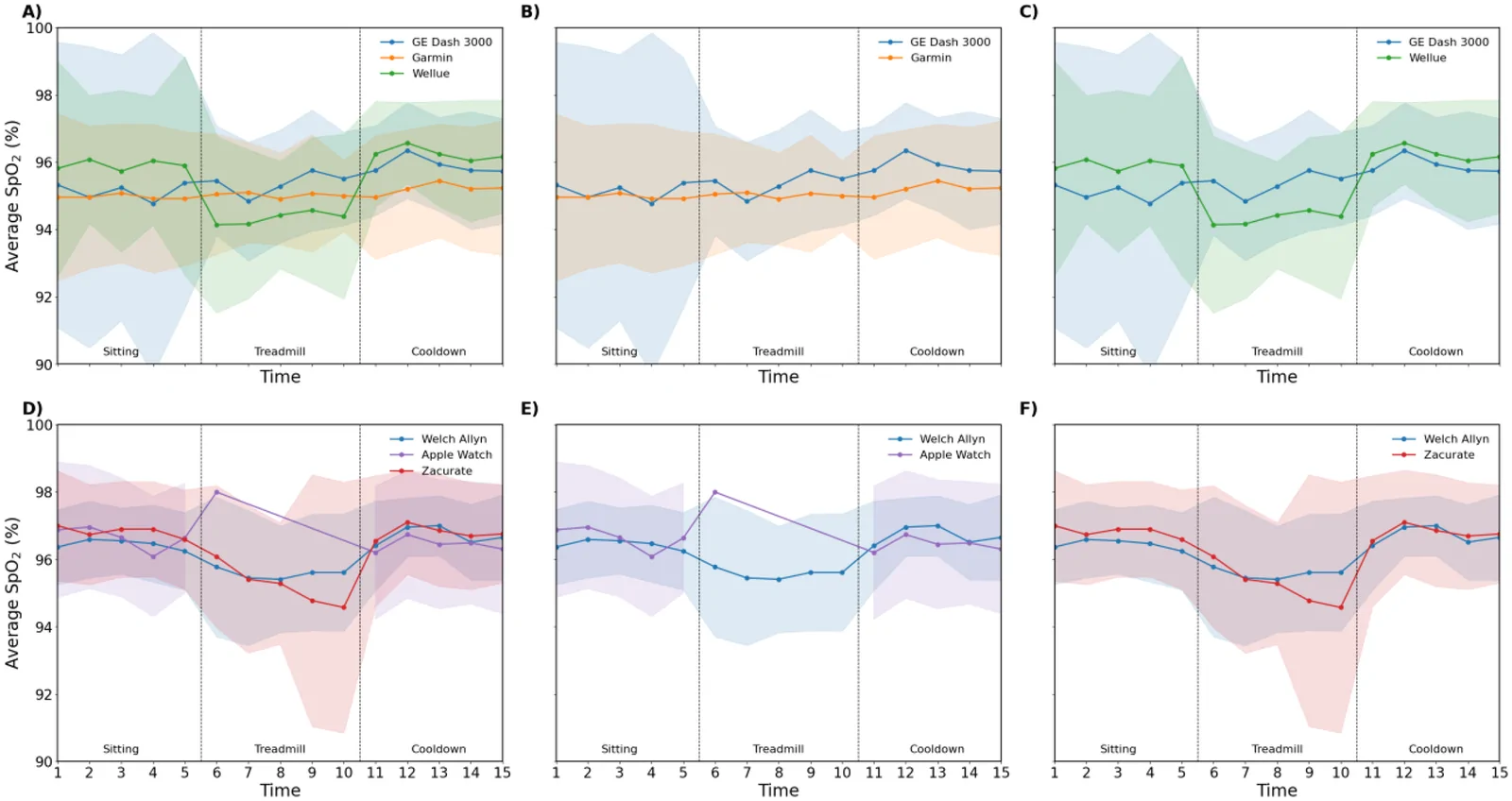
Mean SpO₂ measurements over time across the standardized testing protocol in 49 participants. The top row shows the GE Dash 3000 testing round: **(A)** all three devices, **(B)** Garmin versus GE Dash 3000, and **(C)** Wellue versus GE Dash 3000. The bottom row shows the Welch Allyn testing round: **(D)** all three devices, **(E)** Apple Watch versus Welch Allyn, and **(F)** Zacurate versus Welch Allyn. Each line represents the mean SpO₂ for one device, markers indicate mean values at each time point, and shaded regions represent ±1 standard deviation. A total of 4,118 valid device-level measurements contributed to the figure. Vertical dashed lines indicate transitions between sitting, treadmill, and cooldown. Missingness is summarized in Table 3 and Supplementary Table 1.

### Condition-dependent changes in agreement

Device-specific contrasts were used to evaluate changes in signed and absolute error between activity conditions (Table 6). For Wellue, signed error differed significantly between cooldown and treadmill conditions by 1.37 percentage points (95% CI 0.89 to 1.85; Holm-adjusted *p* < 0.001). Absolute error also differed between cooldown and treadmill by −0.62 percentage points (95% CI −1.06 to −0.19; Holm-adjusted *p* = 0.048). No Garmin condition contrasts were statistically significant after multiple-comparison correction. Apple Watch treadmill-related contrasts were not interpreted because of insufficient treadmill measurements. No Zacurate condition contrast remained statistically significant after Holm correction.

**Table 6:** Device-specific changes in measurement error between activity conditions. The table includes the statistically significant condition effects and selected near-threshold contrasts; no other device-specific condition contrasts remained significant after Holm correction.

Device	Outcome	Condition contrast	Estimate (95% CI)	Holm-adjusted p
Wellue	Signed error	Cooldown vs. Treadmill	1.37 (0.89, 1.85)	<0.001
Wellue	Absolute error	Cooldown vs. Treadmill	−0.62 (−1.06, −0.19)	0.048
Wellue	Signed error	Treadmill vs. Sitting	−1.80 (−3.06, −0.54)	0.051
Apple Watch	Signed error	Cooldown vs. Sitting	−0.46 (−0.81, −0.11)	0.081
Zacurate	Signed error	Treadmill vs. Sitting	−0.73 (−1.27, −0.18)	0.081
Zacurate	Absolute error	Treadmill vs. Sitting	0.60 (0.16, 1.05)	0.071

### Skin pigmentation analyses

No device-specific association between skin pigmentation and either signed error or absolute error remained statistically significant after correction for multiple comparisons (Table 7). Distributions of absolute SpO₂ error across skin pigmentation groups are presented in Figure 4. Continuous ITA analyses did not identify significant pigmentation-associated effects across the estimable device and activity-condition combinations. Secondary analyses using grouped Fitzpatrick skin types I–II, III–IV, and V–VI produced similar results, with no device-specific contrasts remaining significant after Holm correction. At the device level, Fitzpatrick analyses of absolute error for the Apple Watch (*p* = 0.054) and Garmin (*p* = 0.061) approached but did not reach statistical significance (Table 7). These findings were therefore treated as exploratory.

**Table 7:** Device-level tests of overall associations between skin pigmentation and measurement error.

Device	Outcome	Continuous ITA p	Fitzpatrick p
Garmin	Signed error	0.178	0.716
Garmin	Absolute error	0.530	0.061
Wellue	Signed error	0.272	0.708
Wellue	Absolute error	0.446	0.353
Apple Watch	Signed error	0.823	0.308
Apple Watch	Absolute error	0.382	0.054
Zacurate	Signed error	0.208	0.867
Zacurate	Absolute error	0.234	0.152

**Figure 4:**
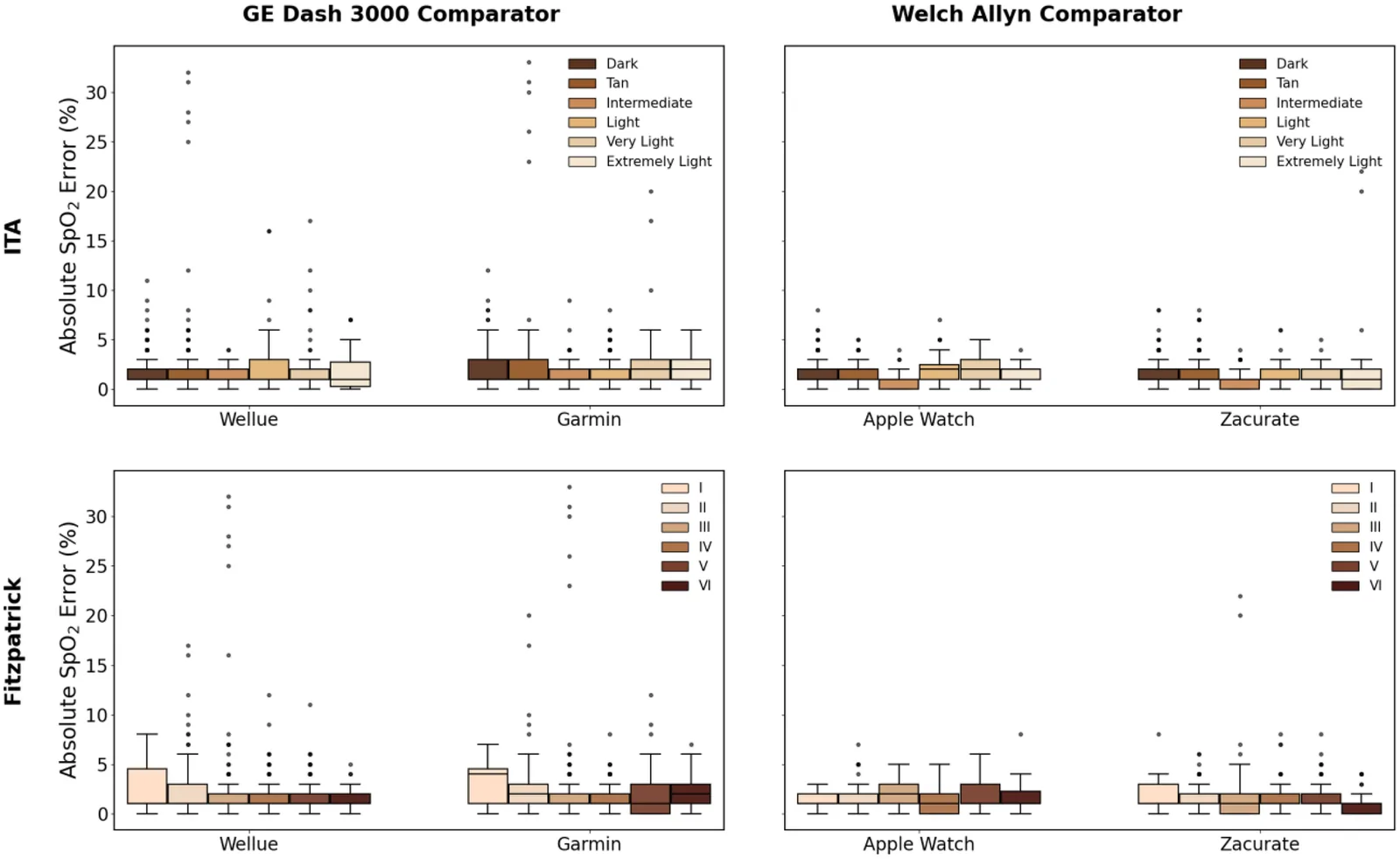
Absolute SpO₂ error across skin pigmentation groups (*n* = 49). Wellue and Garmin were compared with the GE Dash 3000 (left), while Apple Watch and Zacurate were compared with the Welch Allyn monitor (right). Skin pigmentation was characterized using ITA categories (top) and Fitzpatrick skin types I–VI (bottom). Boxplots show the distribution of individual absolute SpO₂ errors within each group.

Sensitivity analyses additionally adjusting for age, BMI, and sex produced estimates that were essentially unchanged from the primary models, indicating that these covariates did not materially alter the observed pigmentation-related findings.

### Bland–Altman agreement

Repeated-measures Bland–Altman analysis was performed separately for each synchronized consumer–comparator pair and activity condition. Corresponding Bland–Altman plots are shown in Figures 5, 6. Mean bias and 95% limits of agreement varied across devices and conditions (Table 8).

**Figure 5:**
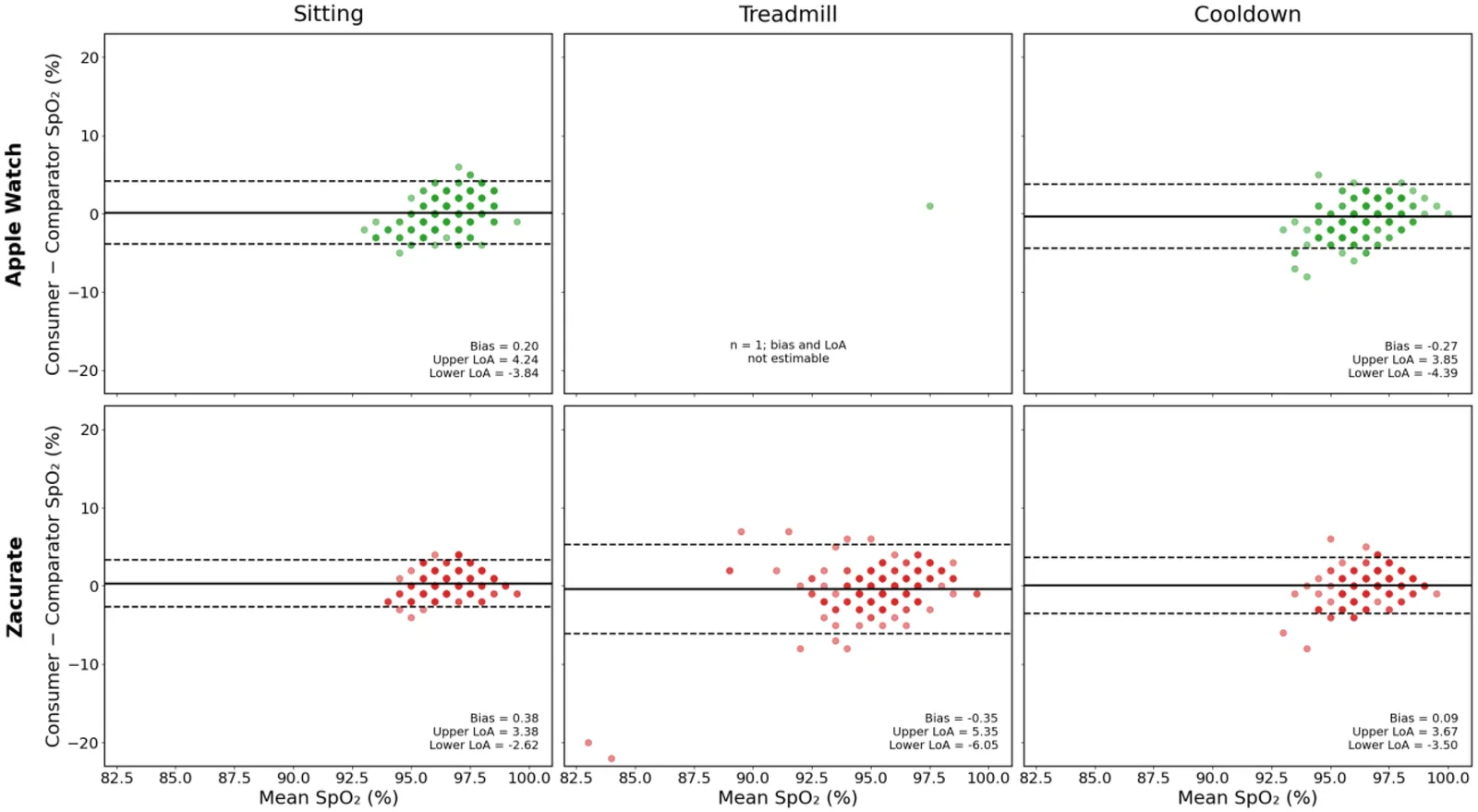
Repeated-measures Bland–Altman plots comparing Apple Watch and Zacurate with the Welch Allyn across sitting, treadmill, and cooldown conditions (*n* = 49; 1,425 paired observations). The top row shows Apple Watch versus Welch Allyn and the bottom row shows Zacurate versus Welch Allyn. Solid horizontal lines indicate mean bias, and dashed lines indicate the 95% limits of agreement.

**Figure 6:**
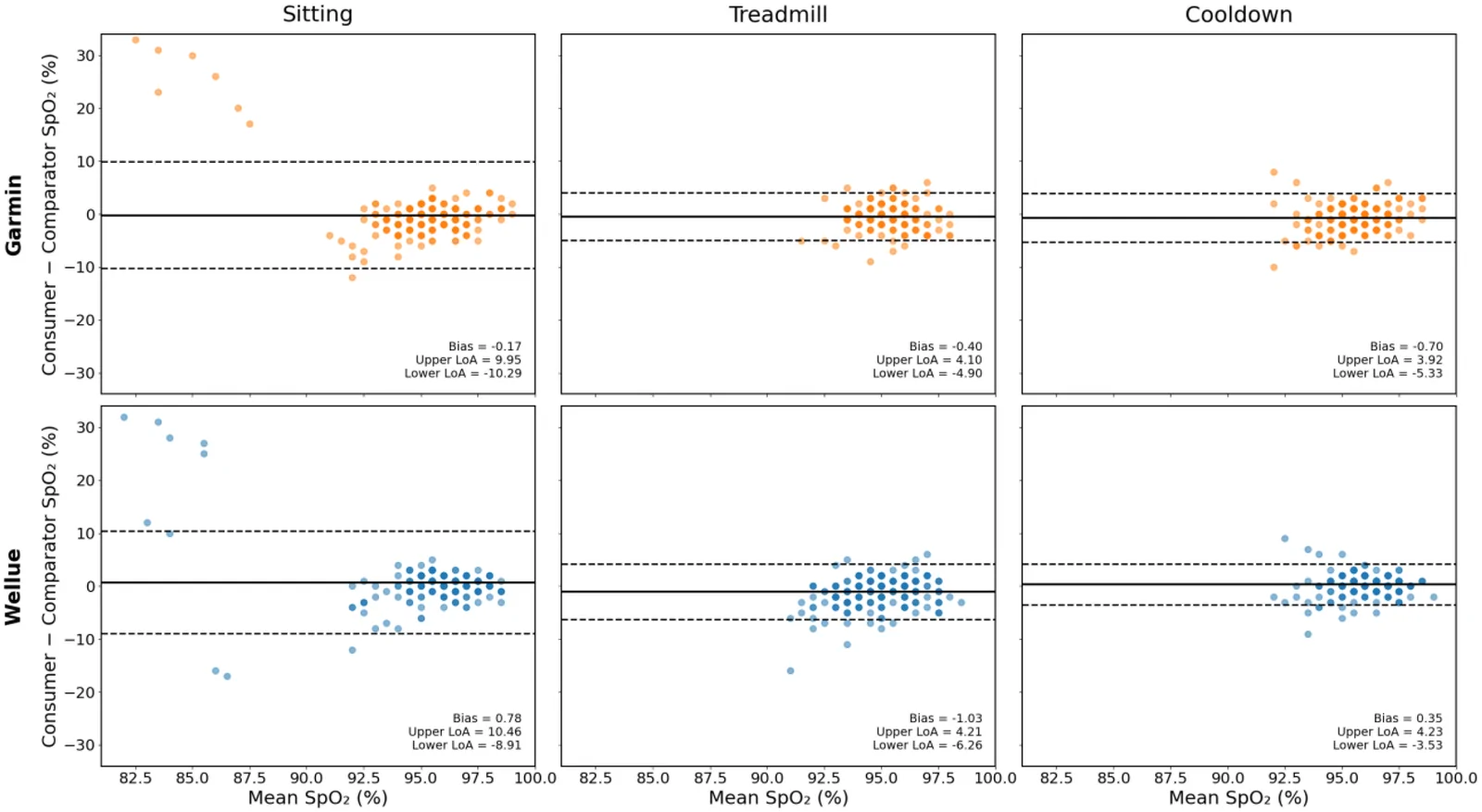
Repeated-measures Bland–Altman plots comparing Garmin and Wellue with the GE Dash 3000 across sitting, treadmill, and cooldown conditions (*n* = 49; 1,423 paired observations). The top row shows Garmin versus GE Dash 3000 and the bottom row shows Wellue versus GE Dash 3000. Solid horizontal lines indicate mean bias, and dashed lines indicate the 95% limits of agreement.

**Table 8:** Repeated-measures Bland–Altman agreement between consumer pulse oximeters and their corresponding clinical comparators across activity conditions. Garmin and Wellue were compared with the GE Dash 3000, while Apple Watch and Zacurate were compared with the Welch Allyn. Mean bias and the lower and upper 95% limits of agreement (LoA) are reported with corresponding 95% confidence intervals. Apple Watch treadmill agreement was not estimable because only one valid synchronized measurement was available.

Device	Condition	N	Mean Bias (95% CI)	Lower LoA (95% CI)	Upper LoA (95% CI)
*GE Dash 3000 comparator*					
Garmin	Sitting	245	−0.17 (−1.17, 1.39)	−10.29 (−14.52, −5.08)	9.95 (3.42, 16.71)
Garmin	Treadmill	202	−0.40 (−0.85, 0.09)	−4.90 (−5.79, −3.94)	4.10 (3.41, 4.80)
Garmin	Cooldown	241	−0.70 (−1.16, −0.26)	−5.33 (−6.09, −4.44)	3.92 (3.17, 4.58)
Wellue	Sitting	245	0.78 (−0.09, 2.09)	−8.91 (−13.11, −3.98)	10.46 (4.49, 16.74)
Wellue	Treadmill	245	−1.03 (−1.51, −0.54)	−6.26 (−7.53, −4.91)	4.21 (3.40, 4.96)
Wellue	Cooldown	245	0.35 (−0.04, 0.74)	−3.53 (−4.32, −2.65)	4.23 (3.39, 5.08)
*Welch Allyn comparator*					
Apple Watch	Sitting	244	0.20 (−0.20, 0.62)	−3.84 (−4.32, −3.25)	4.24 (3.65, 4.77)
Apple Watch	Treadmill	1	Not estimable	Not estimable	Not estimable
Apple Watch	Cooldown	245	−0.27 (−0.69, 0.15)	−4.39 (−5.15, −3.60)	3.85 (3.27, 4.43)
Zacurate	Sitting	245	0.38 (0.07, 0.71)	−2.62 (−2.98, −2.15)	3.38 (2.80, 3.91)
Zacurate	Treadmill	245	−0.35 (−0.82, 0.14)	−6.05 (−8.52, −3.95)	5.35 (3.54, 7.26)
Zacurate	Cooldown	245	0.09 (−0.26, 0.46)	−3.50 (−4.13, −2.74)	3.67 (3.03, 4.27)

For Garmin relative to the GE Dash 3000, limits of agreement were widest during sitting (−10.29 to 9.95 percentage points) and narrower during treadmill (−4.90 to 4.10) and cooldown (−5.33 to 3.92). Wellue similarly showed wider limits during sitting (−8.91 to 10.46) than during treadmill (−6.26 to 4.21) or cooldown (−3.53 to 4.23).

For Apple Watch relative to Welch Allyn, limits of agreement were −3.84 to 4.24 percentage points during sitting and −4.39 to 3.85 during cooldown. Apple Watch treadmill Bland–Altman agreement was not estimable. Zacurate limits of agreement were −2.62 to 3.38 during sitting, −6.05 to 5.35 during treadmill, and −3.50 to 3.67 during cooldown. Mean bias, 95% limits of agreement, and corresponding 95% confidence intervals are reported in Table 8. Intraclass correlation coefficients were generally low across devices and conditions, with the highest agreement observed for Zacurate during treadmill activity (ICC = 0.43, 95% CI 0.21–0.66; Table 9). Apple Watch treadmill ICC was not estimable because of insufficient valid measurements.

**Table 9:** Intraclass correlation coefficients between consumer pulse oximeters and their corresponding clinical comparators across activity conditions. ICCs represent two-way mixed-effects, absolute-agreement, single-measure estimates with 95% confidence intervals. Apple Watch treadmill ICC was not estimable because only one valid synchronized measurement was available.

Device	Condition	N	ICC (95% CI)
*GE Dash 3000 comparator*			
Garmin	Sitting	245	−0.17 (−0.26, 0.33)
Garmin	Treadmill	202	−0.09 (−0.34, 0.20)
Garmin	Cooldown	241	0.03 (−0.21, 0.27)
Wellue	Sitting	245	0.04 (−0.12, 0.76)
Wellue	Treadmill	245	0.07 (−0.14, 0.31)
Wellue	Cooldown	245	0.25 (−0.05, 0.53)
*Welch Allyn comparator*			
Apple Watch	Sitting	244	0.18 (−0.09, 0.41)
Apple Watch	Treadmill	1	Not estimable
Apple Watch	Cooldown	245	0.20 (−0.11, 0.43)
Zacurate	Sitting	245	0.36 (0.11, 0.57)
Zacurate	Treadmill	245	0.43 (0.21, 0.66)
Zacurate	Cooldown	245	0.22 (−0.09, 0.48)

## Discussion

This study evaluated agreement between four consumer pulse oximetry devices and two clinical comparator pulse oximeters across seated rest, treadmill walking, and seated recovery conditions while incorporating both continuous ITA and Fitzpatrick skin type as measures of skin pigmentation. The primary findings were that agreement varied by device and activity condition, with significant negative signed bias observed for Garmin during cooldown and Wellue during treadmill activity. Wellue also demonstrated significant condition-dependent changes in both signed and absolute error between treadmill and cooldown. In contrast, no statistically significant pigmentation-associated effect remained after correction for multiple comparisons using either continuous ITA or grouped Fitzpatrick skin type. Measurement availability also differed substantially by device and condition, particularly for Garmin and Apple Watch during treadmill activity.

The observed condition-dependent differences highlight the importance of evaluating wearable SpO₂ devices under dynamic as well as resting conditions. Wellue demonstrated a significant shift in signed error between treadmill and cooldown, accompanied by a significant reduction in absolute error during cooldown relative to treadmill. Garmin did not demonstrate significant condition-dependent changes after multiple-comparison correction, although measurement availability decreased during treadmill walking. Bland–Altman analyses further demonstrated that the magnitude and spread of consumer–comparator differences varied across devices and conditions (Table 8). The generally low ICCs observed across devices and conditions further indicate limited agreement between consumer devices and their clinical comparators, although interpretation of ICC should consider the relatively narrow, predominantly normoxic SpO₂ range of the study cohort (Table 9). These findings indicate that average bias alone does not fully characterize device performance, because conditions with relatively small mean bias may still exhibit wide limits of agreement.

Measurement availability represented an additional important component of device performance. The GE Dash 3000, Welch Allyn, Wellue, and Zacurate produced complete measurements across all conditions, whereas Garmin availability decreased to 82.4% during treadmill activity. Apple Watch treadmill acquisition was nearly absent, with only 1 of 245 expected measurements available and 48 of 49 participants producing no treadmill readings. This finding should be interpreted primarily as a measurement-availability limitation rather than evidence of systematic SpO₂ over- or underestimation during movement. The Apple Watch Series 6 requires the user to remain stationary during on-demand Blood Oxygen measurements, which is incompatible with continuous treadmill walking and likely contributed to the limited treadmill data obtained in this protocol.

Skin pigmentation was evaluated using both continuous ITA and grouped Fitzpatrick skin type. Although descriptive distributions suggested variability across pigmentation groups, the repeated-measures analyses did not identify statistically significant device-specific associations between pigmentation and either signed or absolute error after Holm correction (Table 7). These findings therefore do not support the conclusion that darker skin pigmentation was associated with greater measurement error in this cohort. However, device-level Fitzpatrick analyses of absolute error for the Apple Watch and Garmin approached statistical significance. Although these findings were not statistically significant, the near-threshold Fitzpatrick results may reflect device-specific variation in performance across pigmentation groups; however, the present data are insufficient to determine whether these patterns represent reproducible effects. These findings should be considered exploratory and should not be interpreted as evidence that larger studies would necessarily identify significant effects.

The absence of statistically significant pigmentation effects in the present study does not eliminate the potential importance of skin pigmentation in optical SpO₂ sensing. Monte Carlo light-transport studies have demonstrated that epidermal melanin can alter photon propagation and pulse modulation at wavelengths used for pulse oximetry (21, 22). Clinical studies have also reported pigmentation-associated differences in pulse oximetry performance (1, 3, 16). The present results instead indicate that any pigmentation-associated effects within this cohort were not sufficiently large or precise to remain statistically significant after accounting for repeated measurements and multiple comparisons. Differences between the present findings and previous reports may reflect device architecture, anatomical placement, participant characteristics, the relatively narrow normoxic SpO₂ range, or limited sample sizes within the lightest and darkest pigmentation groups.

The two clinical pulse oximeters used in this study should be interpreted as clinical comparator devices rather than gold-standard references. Arterial blood gas co-oximetry was not performed, and participants remained predominantly within the normal oxygen saturation range. Accordingly, the present findings characterize agreement between consumer devices and clinical pulse oximeters under primarily normoxic conditions rather than absolute accuracy across the full range of arterial oxygen saturation. The results should therefore not be extrapolated to the detection of clinically significant hypoxemia or to claims regarding missed hypoxemic events or delayed clinical intervention.

The fixed testing sequence represents an additional design limitation. All participants completed the Garmin, Wellue, and GE Dash testing round before the Apple Watch, Zacurate, and Welch Allyn round. Because testing order did not vary between participants, potential order effects such as fatigue, physiological adaptation, changes in peripheral perfusion, or incomplete recovery could not be estimated independently from testing round. To minimize inappropriate cross-round comparisons, analyses were restricted to consumer devices and clinical comparators measured simultaneously within the same testing round. Nevertheless, direct comparisons of performance between the two testing sets should be interpreted cautiously. Future studies should randomize testing order or acquire measurements simultaneously whenever feasible.

Several additional limitations should be considered. An *a priori* sample size calculation was not performed, and the study may therefore have been underpowered to detect smaller device- or pigmentation-related effects. Although the cohort included a broad range of objectively measured ITA values, the grouped Fitzpatrick analysis included only 12 participants in I–II and 11 in V–VI, limiting precision at the pigmentation extremes. The cohort also consisted predominantly of young, healthy adults and excluded participants with diagnosed respiratory disorders. This produced a relatively narrow, primarily normoxic range of SpO₂ values and limits generalizability to older adults and individuals with respiratory disease, cardiovascular disease, impaired perfusion, or clinically meaningful hypoxemia. SpO₂ values were manually recorded, introducing the possibility of transcription error, although measurements were synchronized within each testing round. Finally, the controlled laboratory protocol may not reflect performance during prolonged, unsupervised, or free-living use.

Future studies should incorporate larger prospectively powered cohorts with greater representation across pigmentation levels, randomization of device-testing order, automated synchronized data acquisition, and direct assessment of motion and peripheral perfusion. Inclusion of broader oxygen saturation ranges and comparison with arterial co-oximetry would be necessary to evaluate true measurement accuracy and performance during clinically relevant hypoxemia. Combining experimental evaluation with computational light-transport modeling may also help determine how device geometry, sensor placement, wavelength selection, and tissue optical properties contribute to observed differences in wearable SpO₂ performance.

## Conclusion

This study demonstrated that agreement between consumer wearable pulse oximeters and clinical comparator pulse oximeters varied by device and activity condition under predominantly normoxic conditions. Significant device-specific bias and condition-dependent differences were observed, while no pigmentation-associated effects remained statistically significant after correction for multiple comparisons using either ITA or Fitzpatrick skin type. Because arterial blood gas co-oximetry was not used as the reference standard, these findings reflect agreement rather than absolute measurement accuracy. Future studies incorporating arterial co-oximetry, broader oxygen saturation ranges, and more diverse clinical populations are needed to further evaluate wearable pulse oximeter performance and support the development of more equitable wearable health technologies.

## Data Availability

The datasets presented in this article are not readily available due to data-sharing restrictions stipulated in the study's Institutional Review Board approval. Requests to access the datasets should be directed to Dhruv R. Seshadri, dhs223@lehigh.edu.
